# Behind the veil – exploring the diversity in *Phallus indusiatus* s.l. (Phallomycetidae, Basidiomycota)

**DOI:** 10.3897/mycokeys.58.35324

**Published:** 2019-10-02

**Authors:** Tiara S. Cabral, Bianca DB. Silva, María P. Martín, Charles R. Clement, Kentaro Hosaka, Iuri G. Baseia

**Affiliations:** 1 Instituto Nacional de Pesquisas da Amazônia, Manaus, Amazonas, Brazil Instituto Nacional de Pesquisas da Amazônia Manaus Brazil; 2 Universidade Federal da Bahia, Salvador, Bahia, Brazil Universidade Federal da Bahia, Salvador Bahia Brazil; 3 Real Jardín Botánico-CSIC, Madrid, Spain Real Jardín Botánico-CSIC Madrid Spain; 4 National Museum of Nature and Science, Tsukuba, Ibaraki, Japan National Museum of Nature and Science Tsukuba Japan; 5 Universidade Federal do Rio Grande do Norte, Natal, Rio Grande do Norte, Brazil Universidade Federal do Rio Grande do Norte Natal Brazil

**Keywords:** Amazonia, *atp*6, ITS, Neotropics, nuc-LSU, Phallales

## Abstract

Studies have demonstrated that many cosmopolitan species actually consist of divergent clades that present high levels of morphological stasis throughout their evolutionary histories. *Phallus indusiatus* s.l. has been described as a circum-tropical species. However, this distribution may actually reflect the lack of taxonomic resolution due to the small number of diagnostic morphological characters, which leads to the identification of new records as populations of *P. indusiatus*. Here, we examine the diversity of *P. indusiatus*-like species in Brazilian Amazonia. We show a clear congruence between detailed morphological data and ITS, nuc-LSU and *atp*6 based phylogenetic analyses and three new species are described within the Brazilian indusiate clade. These results highlight the importance of more detailed investigation, with the inclusion of molecular information, in Neotropical fungi.

## Introduction

The worldwide distribution of fungal species hypotheses has been questioned by modern molecular analyses. Studies have demonstrated that many cosmopolitan species actually consist of divergent clades that present high levels of morphological stasis throughout their evolutionary histories (Mueller et al. 2001, Bickford et al. 2007, Geml et al. 2008, Davis et al. 2014). *Phallus indusiatus* Vent. – also known as the “veiled lady” mushroom – has been described as a circum-tropical species, with records for South and Central America (Dennis 1960, Saénz and Nassar 1982, Leite et al. 2007, Cheype 2010), Mexico (Guzmán et al. 1990), Africa (Dissing and Lange 1962, Dring 1964, Demoulin and Dring 1975, Dring and Rose 1977, Desjardin and Perry 2015), Asia (Dennis 1953, Liu 1984, Hosaka 2010) and Australia (Smith 2005). For some groups of fungi, spore dispersal mechanisms may support the idea of transoceanic dispersal connecting geographically isolated populations (Halling et al. 2008, Hosaka et al. 2008). However, the current distribution of *P. indusiatus* may actually reflect the lack of taxonomic resolution due to the small number of diagnostic morphological characters, which leads to the identification of new records as populations of *P. indusiatus*. The insect-dependent mechanism of spore dispersal may also have played an important role in determining the current distribution of *P. indusiatus*.

As in phalloid fungi in general, few morphological characters are available to delimit species in *Phallus*. In addition, most of the widely used diagnostic characters – such as colour and sizes – show high plasticity, another factor that may lead to misidentifications and mask the real diversity within the genus (Kreisel 1996, Calonge 2005). As a consequence of these taxonomic uncertainties, a great number of synonyms are reported for several species of this clade. *Phallus indusiatus* is an emblematic example, where at least nineteen synonyms and several distinct forms have been described (Lloyd 1909, Liu 1984, Guzmán et al. 1990, Kreisel 1996, Calonge 2005, Das et al. 2007, Cheype 2010).

Due to lack of resolution when using morphological characters to identify *Phallus* species, we believe that several specimens that have been identified as *P. indusiatus* might actually consist of independently evolving entities. In fact, some new species with minimal, yet noticeable morphological differences from *P. indusiatus*, have been proposed. For instance, *P. serrata* H.L. Li, L. Ye, P.E. Mortimer, J.C. Xu & K.D. Hyde, described for China, differs by the meshes of the indusium with serrate edges (Li et al. 2014); *P. echinovolvatus* (M. Zang, D.R. Zheng and Z.X. Hu) Kreisel has a whitish volva with mycelioid projections on the surface (Zang et al. 1988); and *P. flavidus* Kreisel & Hausknecht, described for the Seychelles, has yellowish pigments on the receptacle and indusium (Kreisel and Hausknecht 2009). Some of these species were described with the support of molecular analyses, which reinforces the importance of this kind of analysis to resolve these taxonomic uncertainties. At least three species resembling *P. indusiatus* were described for Brazil: *Phallus moelleri* Lloyd, *Dictyophora callichroa* Möller and *Dictyophora phalloidea* Desv. In the original descriptions, they present some inherent characteristics that distinguish them from *P. indusiatus*, such as the above-ground development of the volva in *D. phalloidea* and the orange receptacle and pinkish receptacle apex in *D. callichroa* (Möller 1895). Lloyd (1909) described *P. moelleri* based on a Brazilian species and synonymised *D. callichroa* with it. All three species are now considered synonyms of *P. indusiatus* by some authors and Index Fungorum (Lloyd 1909, Fischer 1928, Saénz and Nassar 1982, Calonge 2005, Kreisel and Hausknecht 2009).

*Phallus indusiatus* was described by Étienne Pierre Ventenat in 1798, based on a specimen from Suriname. In 1809, Desvaux created a new genus, *Dictyophora* Desv., mainly characterised by the presence of an indusium, a skirt-like structure that expands from the receptacle towards the ground. Ventenat’s species was transferred to *Dictyophora* and named *D. indusiata* (Vent.) Desv. Kreisel (1996) considered that the importance of an indusium for the taxonomy of the genus was overestimated, hence he downgraded *Dictyophora* to a section of *Phallus*. More recently, with the introduction of molecular data to the systematics and taxonomy of fungi, studies have shown that the indusium is a recurrent character, which independently emerged several times during the evolution of the group (Hosaka et al. 2006, Cabral et al. 2012, Marincowitz et al. 2015, Trierveiler-Pereira et al. 2017). Today, *P. indusiatus* Vent. is the valid name for Ventenat’s species. *Phallus indusiatus* is widespread in Brazil, with records from four of the six Brazilian biomes (Magnago et al. 2013), but information concerning its diversity and distribution is still incomplete.

In this study, we examined the diversity of *P. indusiatus*-like species in Brazilian Amazonia. We show a clear congruence between detailed morphological data and DNA-based phylogenetic analyses and three new species are described within the Brazilian indusiate clade. These results highlight the importance of more detailed investigation, with the inclusion of molecular information, in Neotropical fungi.

## Material and methods

### Morphological data

**Figure 1.**
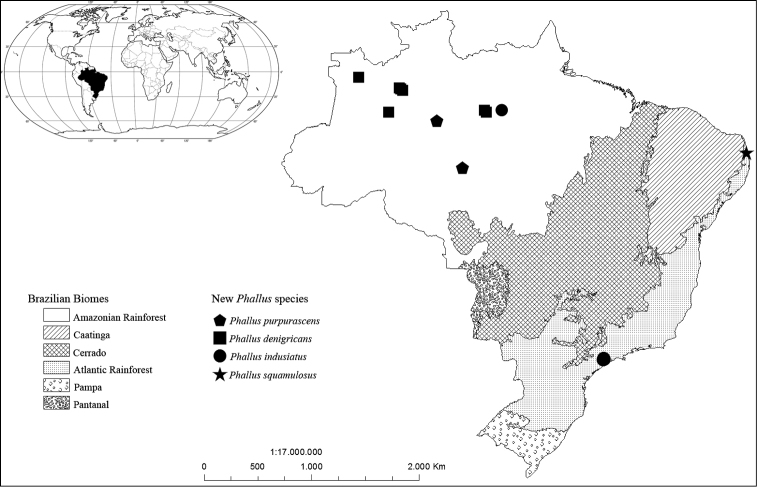
Currently known distributions of the *Phallus* species described in this study. Highlighted areas are the Brazilian Biomes (IBGE 2012): Amazonian Rainforest, Cerrado, Caatinga, Atlantic Rainforest, Pantanal and Pampa.

Specimens of *Phallus* sp. with white indusium were collected during the rainy seasons of 2013 to 2015 in various areas of the Amazon Rainforest domain (Figure 1). We included in the analyses four additional specimens attributed to *P. indusiatus* borrowed from the Herbarium of the Instituto de Botânica (São Paulo) and the Universidade Federal de Rio Grande do Norte-Fungos, which were collected in various areas of the Atlantic Rainforest domain. Other *Phallus* species were included in the molecular analysis to increase taxon coverage, both from GenBank and newly sequenced specimens from the Paleartic-Oriental region (Suppl. material 1: Table S1). Species were morphologically described based on fresh and dried material. Macroscopic characters were described based on field notes and photographs, while microscopic details were obtained by mounting slides with fragments from different layers and structures of dried basidiome in 5% potassium hydroxide (KOH) and/or stained with Congo red dye. We followed the specific literature for species identification (Lloyd 1909, Kreisel 1996, Calonge 2005, Kreisel and Hausknecht 2009) and colours were described following Küppers (1979).

### DNA extraction, amplification and sequencing

DNA extraction followed Hosaka (2009). The nuclear ribosomal ITS and nuc-LSU regions, as well as mitochondrial *atp*6 region, were amplified using previously described primers and protocols (Vilgalys and Hester 1990, White et al. 1990, Kretzer and Bruns 1999). DNA fragments were visualised in 1% agarose gel stained with GelRed™ (Biotium) under UV light. The fragments were purified using Ilustra ExoProStar (GE Healthcare) and then sequenced using the Big Dye Terminator Cycle Sequencing Kit (Applied Biosystems) with the same primer pairs. After sequencing, some ITS electropherograms presented double peaks; in order to resolve these, the ITS PCR fragments were cloned following Marincowitz et al. (2015). All ribotypes were included in the phylogenetic analyses.

### Molecular phylogenetic analyses

We submitted each sequence to a BLAST search to identify the closest relatives and to check for possible contamination. The closest sequences resulting from the BLAST search and sequences with genus names of *Phallus* or *Dictyophora* were retrieved from GenBank and added to the dataset. All sequences were aligned and manually edited with Geneious R6.1 (Biomatters Ltd.). Two analyses were run, one for the ITS dataset (ITS) and the other with ITS, nuc-LSU and *atp*6 concatenated matrix (CONC). The ITS final aligned matrix contained 618 positions, while the concatenated matrix contained 1896 positions (571 for ITS, 794 for nuc-LSU and 529 for *atp*6). These two matrices were analysed separately. Based on a previous phylogeny (Trierveiler-Pereira et al. 2014), species of the genus *Mutinus* were chosen as outgroups. Maximum Parsimony (MP) analyses were performed with PAUP* (Swofford 2003) using heuristic searches with the TBR branch-swapping algorithm; the initial tree was obtained by stepwise addition of random additional sequences repeated 100 times and 1000 replicates as bootstrap (bs) settings. For Bayesian analysis (BA), the substitution model of evolution was chosen with MrModelTest (Nylander 2004). The analyses were run in MrBayes 3.2.6, as follows: two parallel runs were executed with four incrementally-heated simultaneous MCMC simulations over 5 million generations, with trees sampled every 1000 generations. The consensus trees were reconstructed with the remaining trees after the burn-in stage, which was defined based on the average standard deviation of split frequency values. The confidence values were estimated with posterior probabilities (pp). Trees were visualised and edited in FigTree version 1.4.2. All data are available in TreeBASE under ID 21524.

## Results

A total of 19 recently collected specimens of *Phallus* spp. with white indusium were studied, 15 of which were collected in Brazilian Amazonia, while four other specimens were collected from the Brazilian Atlantic Rainforest (SP and UFRN-Fungos herbaria) (Figure 1). Additionally, we obtained sequences from 21 *Phallus* specimens from Japan, Russia, Vietnam and Thailand. The collection localities, herbarium vouchers and GenBank accession numbers can be found in the Suppl. material 1: Table S1, as well as in species descriptions.

### Phylogenetic analyses

**Figure 2.**
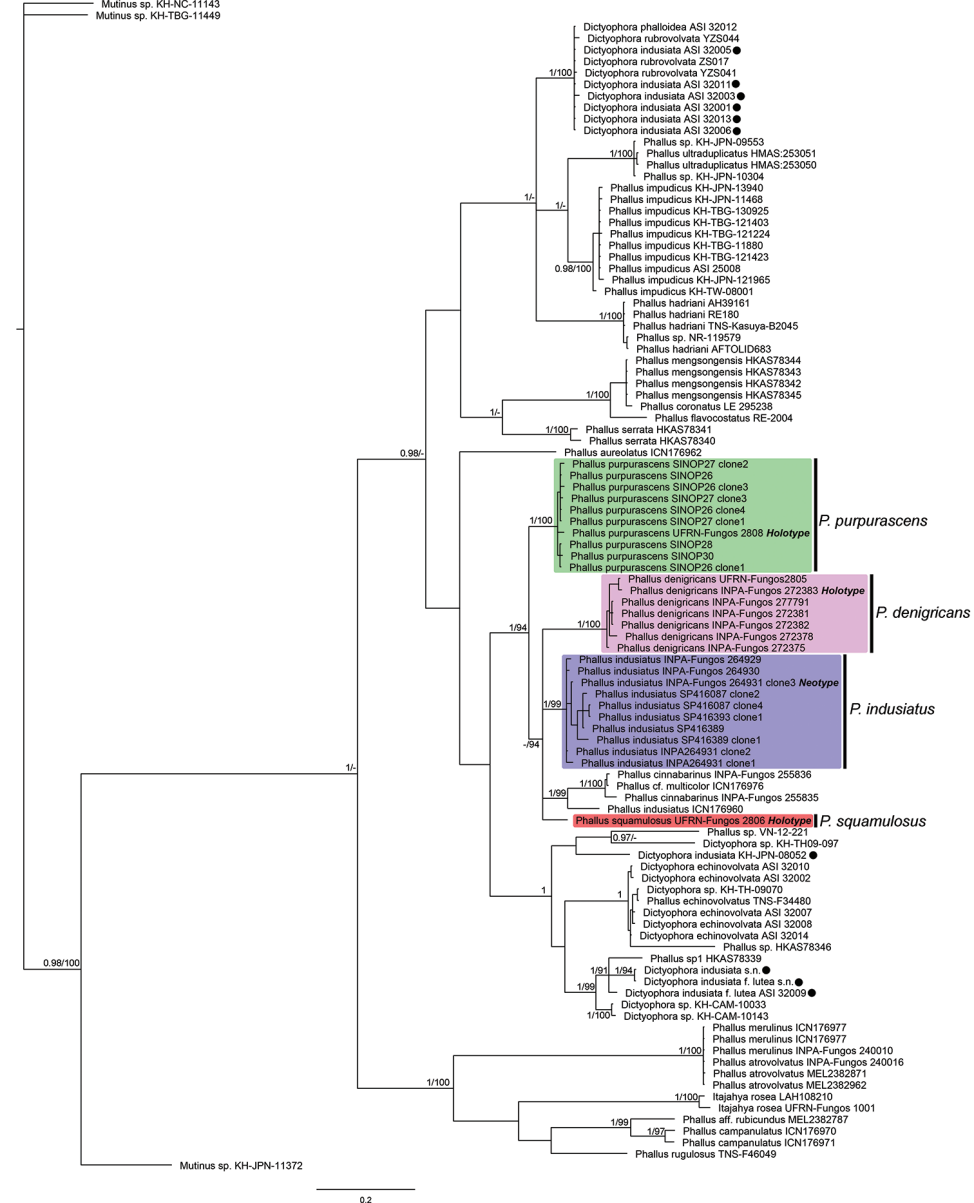
Phylogenetic tree obtained by Bayesian analysis with ITS. Brazilian clades corresponding to the new species and *P. indusiatus* are indicated (the holotype of each species is in bold). Posterior probabilities and bootstrap values are on the nodes (pp/bs), values of pp < 0.95 and bs < 90 are not shown. The black dots indicate specimens under *Phallus indusiatus* deposited in GenBank and downloaded for this study.

**Figure 3.**
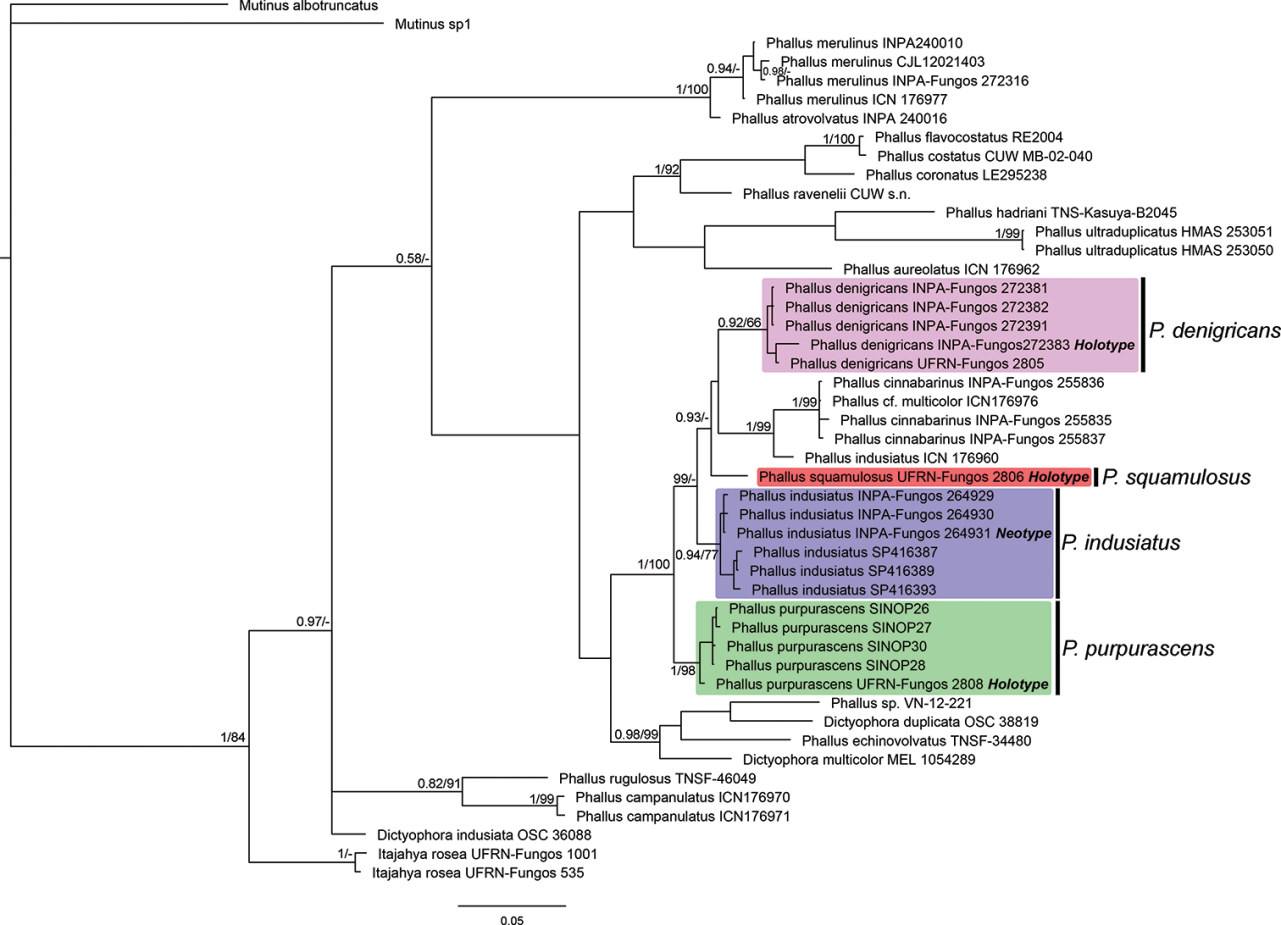
Phylogenetic tree obtained by Bayesian analysis with concatenated data (ITS, nuc-LSU and *atp*6). Brazilian clades corresponding to the new species and *P. indusiatus* are indicated (the holotype of each species is in bold). Posterior probabilities and bootstrap values are on the nodes (pp/bs), values of pp < 0.95 and bs < 90 are not shown (except for *P. denigricans* clade).

We obtained 95 sequences, amongst which 54 were ITS, 19 were nuc-LSU and 22 were *atp*6 (Suppl. material 1: Table S1). The ITS final aligned matrix contained 618 positions, while the concatenated matrix contained 1896 positions (571 for ITS, 795 for nuc-LSU and 530 for *atp6*). Maximum Parsimony and Bayesian analyses with both matrices (ITS and CONC) resulted in trees with the same intraspecific relationships, but with different topologies (Figures 2, 3; MP trees in Suppl. material 2: Figures S1, S2). For Maximum Parsimony analysis, of the 618 positions from the ITS matrix, 382 were informative and resulted in a most parsimonious tree with 2006 steps (CI = 0.458, RI = 0.859, RC = 0.394), while of the 1896 positions from the CONC matrix, 502 were informative and resulted in a most parsimonious tree with 1097 steps (CI = 0.547, RI = 0.709, RC = 0.388). In all of the phylogenetic trees obtained in this study, the Brazilian specimens of *Phallus* grouped together (ITS: pp = 1, bs = 94%; CONC: pp = 1, bs = 100%). This clade can be divided into six groups, which correspond to the four morphospecies identified and described here (coloured clades on Figures 2, 3), *Phallus cinnabarinus* (W.S. Lee) Kreisel found in Amazonia (Cabral et al. 2015) and one specimen from southern Brazil (*P. indusiatus* ICN 176960), for which we do not have morphological information. Sequences under the name *P. indusiatus* (and *D. indusiata*) retrieved from GenBank, all from Asia (China and Japan), as well as those collected by us in this study, form a paraphyletic clade with intercontinental disjunct distributions. Based on morphological similarities and the geographical proximity to the type locality (Suriname) of the Amazonian specimens collected and supported by the molecular data, one Brazilian clade (blue on Figures 2, 3) corresponds to *P. indusiatus* sensu stricto.

## Taxonomy

### Phallus denigricans

Taxon classificationFungiPhallalesPhallaceae

T.S.Cabral, B.D.B.Silva & Baseia sp. nov.

88BA0BA5-D8F8-56AA-8F5F-025EB276DDDA

824632

Figure 4

#### Diagnosis.

This species is characterised by the campanulate receptacle slightly constricted at the base, pale yellow, reticulated, with a prominent apical pore, epigeous development of basidiome, volva varying from white to dark brown and spores up to 4.6 × 2.5 µm.

#### Holotype.

BRAZIL. Amazonas: São Gabriel da Cachoeira, Itacoatiara Mirim Community (0.304167S, 66.8403W), 1 April 2013, Komura DL (INPA-Fungos 272383). GenBank accessions: MG678486 (ITS), MG678455 (nuc-LSU), MG678541 (*atp*6).

Immature basidiomes not observed. Fresh expanded basidiome 98 mm high. Receptacle [25] 26 × 19 [25] mm, campanulate, but slightly constricted at the base, with a prominent apical pore, deeply reticulated surface. Pseudostipe [81] 54 × 10 [22] mm, cylindrical, spongy, white (N__00__A_00_M_00_); pseudoparenchymatous, composed of globose to elongate-ovoid cells, [20.5] 18.5–65.5 [60.8] × [17.5] 19–52.5 [51.2] µm, hyaline. Indusium poorly developed, extending to 2/3 of pseudostipe, white (N_00_A_00_M_00_), 53 mm in length, attached to the apex of the pseudostipe, polygonal to irregular meshes up to 13 × 8 mm. Volva epigeous, white (N_00_A_00_M_00_) in some specimens to dark brown (N_60_A_60_M_50_) in others, with smooth surface or sometimes with small hyphae projections on surface; formed by filamentous hyphae, septate, branched, hyaline, clamp connections present, [2.5] 1.8–5 [3.5] μm diameter, with inflated ends up to 15.5 μm diameter. Rhizomorphs composed of at least two types of hyphae: filamentous thin-walled hyphae, with clamp connections; and thicker hyphae (7–16 µm) that seem to communicate with each other by pores on the inflated tips. Crystals in globose cells were found distributed amongst the hyphae of volva and rhizomorphs of some of the white volva species, measuring 8.2–11.5 × 6.8–10.6 μm. Gleba olive brown (N_99_A_50_M_10_), mucilaginous. Basidiospores elongated, smooth, 3.6–4.6 × 2.2–2.5 µm, hyaline in 5% KOH.

**Figure 4.**
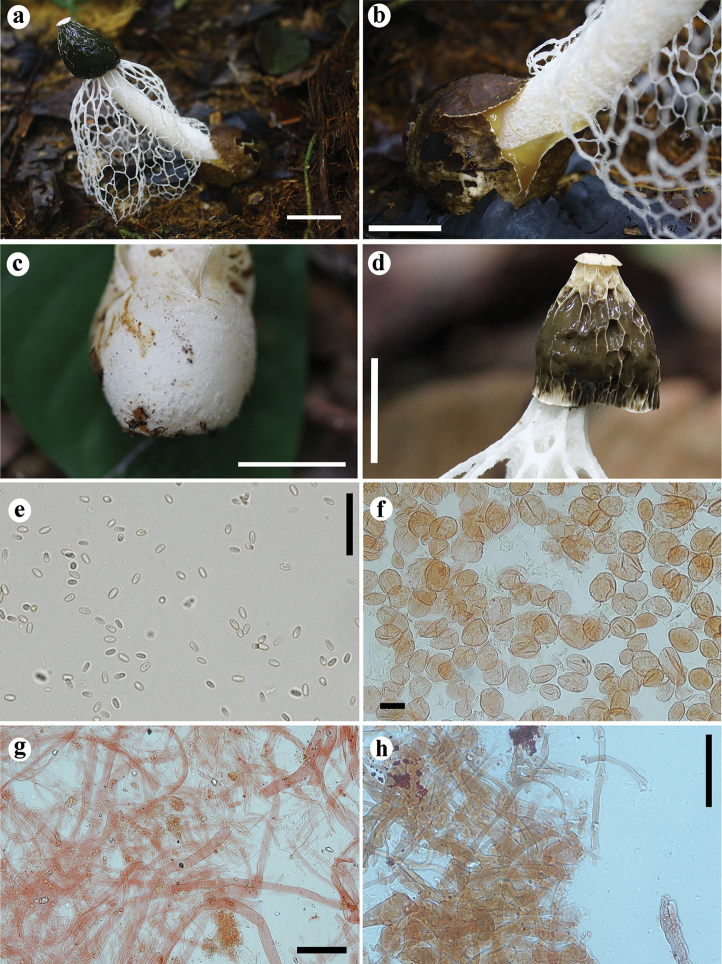
*Phallus denigricans* UFRN-Fungos 2805, holotype. **A** Basidiome **B** blackish and smooth volva in detail **C** white volva with projections **D** receptacle with a prominent pore **E** spores **F** pseudoparenchymatous hyphae of pseudostipe **G** hyphae from rhizomorphs **H** hyphae from volva. Scale bars: 20 mm (**A–D**), 20 µm (**E**), 40 µm (**F–H**).

#### Habitat and distribution.

On soil, in a fragment of upland old-growth forest. So far restricted to the Brazilian Atlantic and Amazon forests, found in the municipalities of Barcelos, Parintins, São Gabriel da Cachoeira and Maraã (State of Amazonas, Brazil); and Natal (State of Rio Grande do Norte).

#### Etymology.

with reference to the volva becoming blackish.

#### Other specimens examined (paratypes).

Brazil. Amazonas: Maraã, Reserva de Desenvolvimento Sustentável do Amanã, Ubim Community (2.50500S, 64.66039W), 15 February 2014, Cabral TS (UFRN-Fungos 2805). Barcelos, Bacabal Community (0.49004S, 62.93089W), 7 April 2015, Cabral TS (INPA-Fungos 277791). Parintins, Açaí Community (2.62665S, 56.54041W), 5 March 2015, Cabral TS (INPA-Fungos 272375); 6 March 2015 (INPA-Fungos 272378); Barcelos, Bacabal Community (0.49004S, 62.93089W), 7 April 2015, Cabral TS (INPA-Fungos 272381, INPA-Fungos 272382). Rio Grande do Norte: Natal (6.305093S, 35.361112W), 10 September 2005, Barbosa MMB (UFRN-Fungos 417).

#### Notes.

*Phallus flavidus* Kreisel & Hauskn. could be comparable with *P. denigricans* by the conical receptacle and the indusium size; however, *P. flavidus* has smaller spores (up to 3.6 × 1.8 µm), the surface of the volva is light grey with an orange flush and the indusium is cream to yellow (Kreisel and Hausknecht 2009). *Phallus impudicus var. obliteratus* (Malençon) Kreisel has a reticulate white receptacle and a rudimentary white indusium; *Phallus denigricans* also has a poorly-developed indusium, but it is very different from P. impudicus var. obliteratus, where the indusium is hidden under the receptacle (Calonge 2005, Kreisel and Hausknecht 2009). *Phallus callichrous* (Möller) Lloyd is a species described from Brazil, with white indusium and differs from *P. denigricans* by having an orange to pink receptacle and reddish-violet rhizomorphs. Recently, another indusiate species was described for Brazil, *Phallus aureolatus*, but it differs from *P. denigricans* mainly by the strongly developed pore and the merulioid surface of the receptacle (Trierveiler-Pereira et al. 2017), in addition to its different phylogenetic placement (Figures 2, 3). *Phallus echinovolvatus* (M. Zang, D.R. Zheng & Z.X. Hu) Kreisel is another white-indusiate species, characterised mainly by the volva covered with echinulate hyphae projections; in *P. denigricans*, hyphae projections on the volva surface can also be found in some specimens, but they are smaller than in *P. echinovolvatus* (Zang et al. 1988). In *P. indusiatus*, the receptacle is campanulate, the immature basidiome is hypogeous, so that the volva is buried under the ground when the basidiome is fully developed, the indusium is completely developed reaching the ground and the volva and rhizomorphs have pinkish pigments (Ventenat 1798). On the other hand, in *P. denigricans* the campanulate receptacle is constricted at the base, the basidiome has a completely epigeous development, the indusium is poorly-developed reaching only 2/3 of the basidiome and the rhizomorphs and volva are white to brownish.

It is not rare to find *Phallus* specimens with a blackish volva; recently, a new species was described, *P. fuscoechinovolvatus* (Song et al. 2018), but it is quite different from *P. denigricans* mainly by the strongly echinulated volva. *Phallus merulinus* (Berk.) Cooke and *P. atrovolvatus* Kreisel & Calonge are very similar, differing by the volva colour – that is black in *P. atrovolvatus* and white in *P. merulinus* – and the habitat (Calonge 2005). In our ITS phylogenetic analyses (Figure 3), specimens identified as *P. atrovolvatus* and *P. merulinus* grouped together in the same clade, indicating a possible identity between these two species. However, no type material was analysed here, which prevents a reliable determination of the species boundaries between *P. atrovolvatus* and *P. merulinus*. Similarly, in *P. denigricans*, we found specimens with white and pale white to brownish volva all grouping in the same clade in phylogenetic trees (Figures 2, 3). This suggests that the volva colour might change due to the soil properties or with the maturity of the basidiome. Therefore, this specific characteristic – pale or darker volva – should be carefully analysed before it can be used as a diagnostic character in *Phallus* species.

In both the Bayesian and Maximum Parsimony phylogenetic trees (Figures 2, 3 and Suppl. material 2: Figures S1, S2, specimens of *P. denigricans* grouped in a clade with high support values (ITS tree: pp = 1, bs = 100%), in concordance with morphological data.

### Phallus purpurascens

Taxon classificationFungiPhallalesPhallaceae

T.S.Cabral, B.D.B.Silva & Baseia sp. nov.

DECB0553-2858-52F9-85F8-8DBE325CD3E4

824633

Figure 5

#### Diagnosis.

This species is characterised by its large basidiome (up to 200 mm), the indusium reaching 2/3 of the basidiome, the purplish volva and rhizomorphs and the thimble-like and strongly reticulated receptacle.

#### Holotype.

BRAZIL. Amazonas: Manaus (3.0615S, 60.0111W), 27 February 2014, Cabral TS (UFRN-Fungos 2808). GenBank accessions: MG678487 (ITS), MG678456 (nuc-LSU), MG678542 (*atp*6).

Immature basidiomes whitish (N_60_A_60_M_50_) with purplish pigments (A_10_M_10_C_10_), globose to subglobose, up to 56 × 43 mm, growing gregariously. Fresh expanded basidiome up to 200 mm high. Receptacle up to 45 × 29 mm, thimble-like, flat at the apex with an apical pore; strongly reticulated surface, shallow reticulations up to 3.2 × 1.7 mm, white (N_00_A_00_M_00_). Pseudostipe up to 122 × 21 mm, cylindrical, spongy, white (N_00_A_00_M_00_); pseudoparenchymatous, composed of globose to elongate-ovoid cells, 37–65.5 × 22.5–48 µm, hyaline. Indusium well-developed, extending up to 2/3 of the pseudostipe, white (N_00_A_00_M_00_), up to 100 mm in length, attached to the apex of the pseudostipe; polygonal meshes up to 10 × 5 mm. Volva semi-hypogeous, white (N_00_A_00_M_00_) becoming purplish (A_10_M_10_C_10_) when exposed, with a smooth surface; formed by filamentous hyphae, septate, branched, hyaline, clamp connections present, 3.1–6.6 μm diameter; with crystal deposits in globose cells widely distributed amongst the hyphae, 17.5–38 × 20.5–35.7 μm. Rhizomorphs composed of at least two types of hyphae: filamentous thin-walled hyphae, with clamp connections; and thicker hyphae (3–6.5 µm) that seem to communicate with each other by pores on the inflated tips. Gleba olive-brown (N_99_A_50_M_10_), mucilaginous. Basidiospores cylindrical, smooth, 4.4–5 × 2.5–3.4 µm, hyaline in 5% KOH.

**Figure 5.**
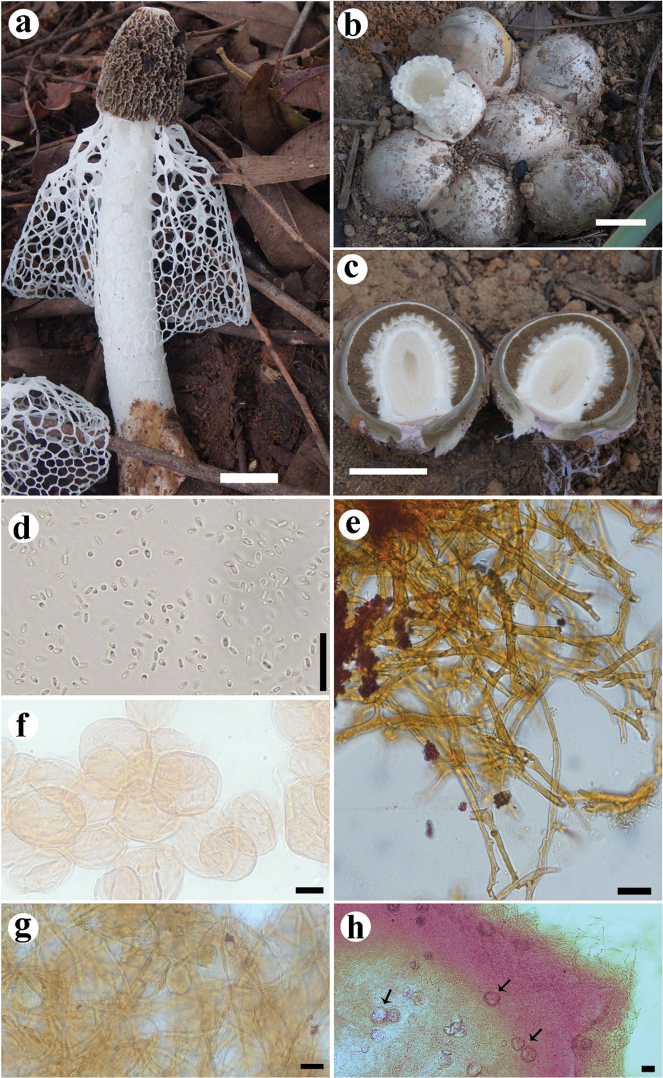
*Phallus purpurascens* SINOP27, paratype. **A** Fresh basidiome **B** gregarious immature basidiome, with purplish pigments on surface **C** longitudinal section of an immature basidiome, showing the purplish volva and rhizomorphs. *Phallus purpurascens* UFRN-Fungos 2808, holotype. **D** Spores **E** rhizomorphs hyphae **F** pseudoparenchymatous hyphae from pseudostipe **G** hyphae from volva **H** crystals in globose cells found on volva. Scale bars: 20 mm (**A–C**), 20 µm (**C–H**).

#### Habitat and Distribution.

on soil, in a fragment of upland secondary forest. It was found in the municipalities of Manaus (State of Amazonas, Brazil) and Sinop (State of Mato Grosso, Brazil).

#### Etymology.

with reference to the volva becoming purple.

#### Other specimens examined (paratypes).

Mato Grosso: Sinop, Parque Florestal de Sinop (11.8359S, 55.5008W), 7 November 2013, Cabral TS (SINOP26, SINOP27, SINOP28, SINOP30).

#### Notes.

This species is the most distinctive amongst our collections, mainly due to its large basidiome, the purplish volva and rhizomorphs and the strongly reticulated receptacle. *Phallus rubrovolvatus* (M. Zang, D.G. Ji & X.X. Liu) Kreisel is one of the largest white-indusiate species (up to 330 mm); it differs from *P. purpurascens* by the deep red volva, the fragile indusium, by larger reticulations on the receptacle and smaller spores (3.7–4 × 2–2.5 µm) (Liu 1984, Calonge 2005). Additionally, in the phylogenetic analysis (Figures 2, 3), *P. rubrovolvatus* does not group with *P. purpurascens*, which confirms their separate identities. *Phallus callichrous* has an orange to pink receptacle, reddish-violet rhizomorphs and orange receptacle with pink margin (Möller 1895, Kreisel and Hausknecht 2009), which differ from the white receptacle, purplish volva and rhizomorphs of *P. purpurascens*; unfortunately, there is little information available for this Brazilian species (Calonge 2005). *Phallus multicolor* (Berk. & Broome) Cooke is similar to *P. purpurascens* in the purplish volva and rhizomorphs, but it differs by the cream to orange indusium and the light yellow pseudostipe (Lloyd 1909, Calonge 2005, Kreisel and Hausknecht 2009). *Phallus indusiatus* differs from *P. purpurascens* by the smaller basidiome, the hypogeous development of the immature basidiome and smaller spores (up to 4.1 × 2.2 µm), the well-developed indusium reaching the ground and the campanulate receptacle with wider reticulations (Ventenat 1798). The phylogenetic analyses show specimens of *P. purpurascens* grouping in a clade with high support values (ITS tree: pp = 1, bs = 100%; CONC tree: pp = 1, bs = 98%), confirming its distinct identity.

*Phallus purpurascens* was found in a fragment of secondary forest, in an extremely threatened area of the Amazonian forest domain in the State of Mato Grosso, Brazil. This state was the second most deforested in Brazil in 2018 (INPE 2018), meaning that species in this area may be suffering the consequences of habitat fragmentation, which is one of the main causes of decline in fungal species (Courtecuisse 2008). Thus, this new species record shows the urgency of cataloguing fungal biodiversity of threatened areas, such as Neotropical forests.

### Phallus squamulosus

Taxon classificationFungiPhallalesPhallaceae

T.S.Cabral, B.D.B.Silva & Baseia sp. nov.

C3FE284E-F729-501D-91B1-343FD3C2EF2F

824634

Figure 6

#### Diagnosis.

This species is characterised by its immature basidiome and volva with a squamous surface, white receptacle with shallow reticulations and a wide pore.

#### Holotype.

BRAZIL. Rio Grande do Norte: Baía Formosa, Reserva Particular do Patrimônio Natural Mata Estrela (6.383307S, 35.000365W), 27 February 2014, Silva BDB (UFRN-Fungos 2806). GenBank accessions: MG678497 (ITS), MG678547 (*atp*6).

Immature basidiomes whitish (N_60_A_60_M_50_), up to 39 × 34 mm, ovoid, with squamous surface. Fresh expanded basidiome up to 95 mm high. Receptacle 20 × 28 mm, campanulate to thimble-like, with a wide apical pore; and a strongly but shallow reticulated surface, reticulations 1.6–2 × 0.8–1.2 mm. Pseudostipe 60 × 12 mm, cylindrical, spongy, white (N_00_A_00_M_00_); pseudoparenchymatous, composed of globose to elongate-ovoid cells, 18–71 × 10.5–35 µm, hyaline. Indusium well-developed, extending to 2/3 of pseudostipe, white (N_00_A_00_M_00_), 44 mm in length, attached to the apex of the pseudostipe; polygonal to rounded meshes up to 6 × 3 mm. Volva epigeous, whitish (N_00_A_00_M_00_) to pale yellow (N_00_C_00_A_30_), with squamous surface; formed by filamentous hyphae, septate, branched, hyaline, clamp connections present, 2.5–4.5 μm diameter. Rhizomorphs whitish (N_00_A_00_M_00_), composed of filamentous thin-walled hyphae, with clamp connections; with crystal deposits in globose cells distributed amongst the hyphae, 15–17.9 × 14–17 μm. Gleba olive-brown (N_99_A_50_M_10_), mucilaginous. Basidiospores elongated, smooth, 3.5–4.4 × 1.8–2.2 µm, hyaline in 5% KOH.

**Figure 6.**
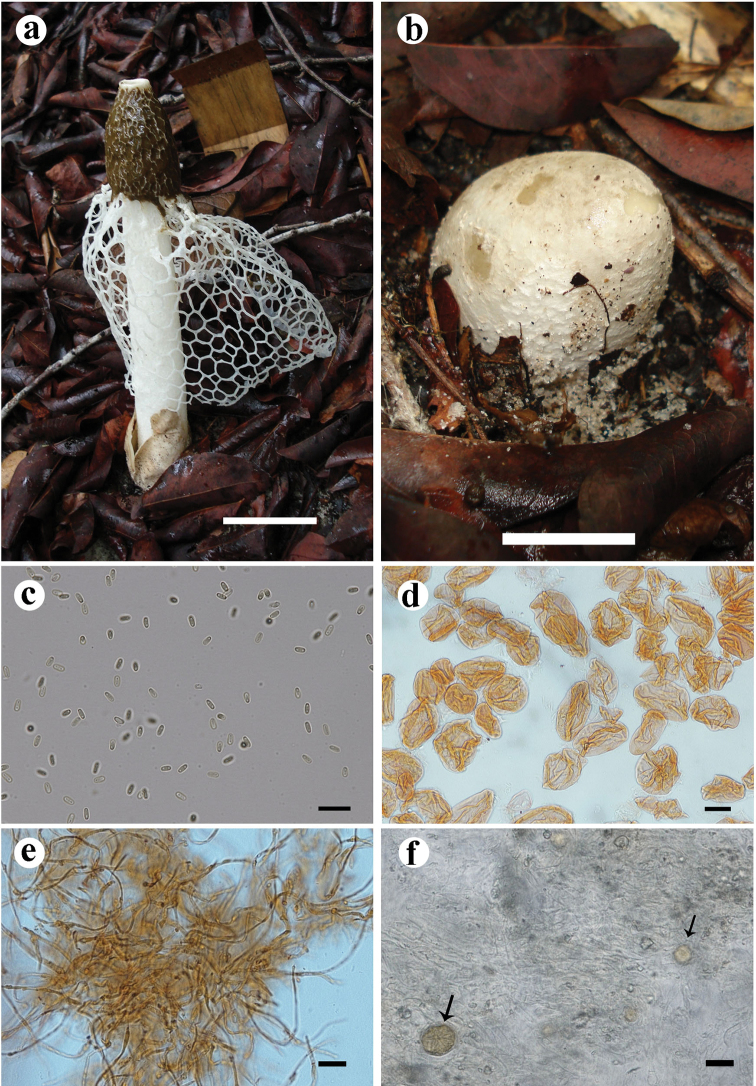
*Phallus squamulosus* UFRN-Fungos 2806, holotype. **A** Fresh basidiome **B** immature basidiome with squamous surface **C** spores **D** pseudoparenchymatous hyphae from pseudostipe **E** hyphae from volva **F** hyphae from rhizomorphs and crystals deposits on globose cells. Scale bars: 20 mm (**A, B**), 20 µm (**C–F**).

#### Habitat and Distribution.

found growing on sandy soil, in a fragment of ombrophilous forest in the Atlantic Rainforest domain.

#### Etymology.

with reference to the volva covered with small scales.

#### Notes.

Only one specimen of this species has been found to date in the northern Atlantic Rainforest domain, but it is quite distinct from other species found in this study. We could not find white-indusiate species records with squamous exoperidium in the available literature. However, *P. duplicatus*, described in Martín and Tabarés (1994), presents an immature basidiome with fine scales on the exoperidium, but this character is not found in other described *P. duplicatus* (Lloyd 1909, Liu 1984, Kreisel and Hausknecht 2009, Kibby and McNeil 2012). Nevertheless, the material described by Martín and Tabarés (1994) differs from *P. squamulosus* mainly by having a conic-campanulate receptacle with crenulate disc on the apex. *Phallus denigricans* presents small hyphae projections on immature exoperidium surfaces of some specimens, but these projections are arranged differently in *P. squamulosus*, where they appear as scales. *Phallus indusiatus* is different from *P. squamulosus* by the campanulate receptacle with a smaller pore and deeper reticulations, the indusium extending to the ground and the immature basidiome that is hypogeous with a smooth surface and pinkish pigments.

### Phallus indusiatus

Taxon classificationFungiPhallalesPhallaceae

Vent., Mém. Inst. Natl. Sci., Sci. Math. 1: 520, 1798

CCFC0D2F-6D5A-5F6A-A12B-8BB8048F3444

245788

Figure 7

≡ Dictyophora indusiata (Vent.) Desv., J. Bot., Paris 2: 92 (1809)
≡ Hymenophallus indusiatus (Vent.) Nees, Syst. Pilze (Würzburg): 251 (1816)
= Dictyophora indusiata f. rosea (Ces.) Kobayasi, J. Jap. Bot. 40: 180 (1965)
= Dictyophora indusiata f. callichroa (Möller) Kobayasi, Trans. Mycol. Soc. Japan 6: 6 (1965)
= Hymenophallus roseus Ces., Atti Accad. Sci. fis. mat. Napoli 8(8): 12 (1879)
= Hymenophallus duplicatus (Bosc) Nees, Syst. Pilze (Würzburg): 251 (1816)
= *Phallus duplicatus* Bosc, Mag. Gesell. naturf. Freunde, Berlin 5: 86 (1811)
= Dictyophora duplicata (Bosc) E. Fisch., in Berlese, De Toni & Fischer, Syll. fung. (Abellini) 7(1): 6 (1888)
= Dictyophora rosea (Ces.) E. Fisch., in Saccardo, Syll. fung. (Abellini) 7(1): 6 (1888)
= Dictyophora phalloidea var. rosea (Ces.) Lloyd, Synopsis of the known phalloids 7: 20 (1909)
= Dictyophora phalloidea var. callichroa (Möller) Lloyd, Synopsis of the known phalloids 7: 20 (1909)
= Dictyophora callichroa Möller, Bot. Mitt. Trop. 7: 129, 148 (1895)
≡ *Phallus callichrous* (Möller) Lloyd, Mycol. Writ. 7: 6 (1907)
= *Phallus indusiatus var. rochesterensis* (Lloyd) Lloyd, Synopsis of the known phalloids 7: 81 (1909)
= *Phallus rochesterensis* Lloyd, Synopsis of the known phalloids 7: 20 (1909)
= Dictyophora phalloidea var. rochesterensis (Lloyd) Sacc. & Trotter, Syll. fung. (Abellini) 21: 460 (1912)
= Dictyophora indusiata f. aurantiaca Kobayasi, Nov. fl. jap. 2: 83 (1938)
= *Phallus indusiatus f. citrinus* K. Das, S.K. Singh & Calonge, Boln Soc. Micol. Madrid 31: 136 (2007)

#### Neotype.

(designated here): BRAZIL. Pará: Belterra, Floresta Nacional do Tapajós, Jamaraqua Community (2.812667S, 55.033083W), 25 March 2014, Cabral TS (INPA-Fungos 264931). GenBank accessions: MG678500, MG678501, MG678502 (ITS); MG678463 (nuc-LSU); MG678550 (*atp*6).

Immature basidiomes not observed. Fresh expanded basidiome 120 mm high. Receptacle 25 × 25 mm, campanulate, with an apical pore, reticulated surface. Pseudostipe 67 × 12 mm, cylindrical, spongy, white (N_00_A_00_M_00_); pseudoparenchymatous, composed of globose to elongate-ovoid cells, 29.5–56.8 × 17.2–44 µm, hyaline. Indusium in full development extending to the ground, white (N_00_A_00_M_00_), 74 mm in length, attached to the apex of the pseudostipe; polygonal to rounded meshes up to 7 × 4 mm, composed of pseudoparenchymatous cells, 31–53.8 × 23.8–41 µm. Volva hypogeous, white (N_00_A_00_M_0_), with pinkish pigments (N_00_M_10_C_00_); outer layer papery, composed of filamentous hyphae, 3.22–6.5 µm, yellowish, septate, with clamp connections; crystal deposits in globose cells distributed amongst the hyphae, 11.5–13.8 × 19.6–22.7 μm. Rhizomorphs composed of filamentous thin-walled hyphae, with clamp connections. Gleba olive-brown (N_99_A_50_M_10_), mucilaginous. Basidiospores elongated, smooth, 3.6–4.1 × 1.5–2.2 µm, hyaline in 5% KOH.

**Figure 7.**
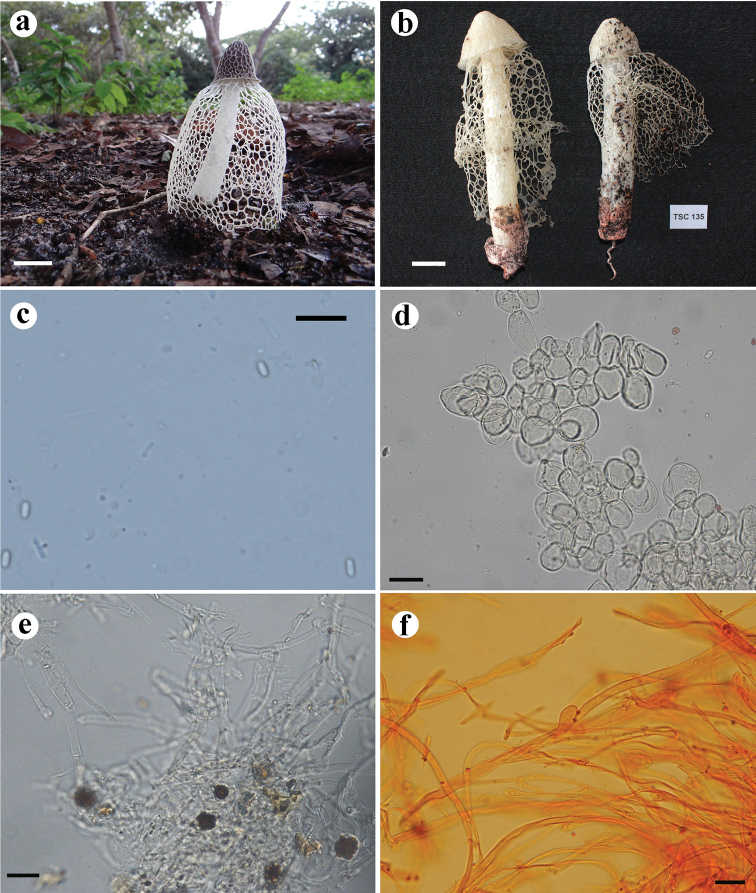
*Phallus indusiatus.* Fresh basidiome of **A** INPA-Fungos 264931 (neotype), and **B** INPA-Fungos 264929, showing the volva with pinkish pigments **C** spores **D** pseudoparenchymatous hyphae from pseudostipe **E** hyphae from volva and crystals deposits on globose cells **F** hyphae from rhizomorphs. Scale bars: 20 mm (**A, B**); 10 µm (**C**); 40 µm (**D**); 20 µm (**E, F**).

#### Habitat and Distribution.

found on sandy soil, in dense old-growth forest. It has a questionable circum-tropical distribution, with records for South and Central America, Mexico, Africa, Asia and Australia, but we believe that the distribution is restricted to South America.

#### Other specimens examined.

BRAZIL. Pará: Belterra, Floresta Nacional do Tapajós, Jamaraqua Community (2.812667S, 55.033083W), 25 March 2014, Cabral TS (INPA-Fungos 264929, INPA-Fungos 264930); São Paulo, Parque Estadual das Fontes do Ipiranga (23.54S, 46.63W), January 2011, Oliveira, J.J.S. (SP416389); March 2011, Ventura, P.O. (SP416393); Capelari, M. (SP416087).

#### Notes.

According to Ventenat’s original description, *P. indusiatus* is characterised by the hypogeous volva, the campanulate and reticulated receptacle and by the indusium reaching the ground. The indusium is white, but it can become reddish as it matures. Ventenat does not give information on the colour of the volva and rhizomorphs, but some authors state that the volva can be light pinkish and rhizomorphs can be pinkish to violet (Calonge 2005, Kreisel and Hausknecht 2009). Our collection presents the same characteristics of the original description and those in the key for indusiate species presented by Kreisel and Hausknecht (2009a); in addition, some of the specimens are from the State of Pará, which is geographically close (about 970 km in a straight line) and with the same forest domain as the type locality (Suriname). Therefore, we believe that the specimens that are nested in the same clade in the phylogenetic trees (Figures 2, 3), all collected in the Brazilian Amazonian and Atlantic rainforests, correspond to *P. indusiatus* sensu stricto. Since Ventenat’s original description does not designate a type specimen and, consequentially, it is not possible to find the original material in herbarium for comparison, we designated here a neotype for *Phallus indusiatus*, in accordance with the provisions of the International Code of Nomenclature for algae, fungi and plants (ICN) (Article 9.8) (Turland et al. 2017).

## Discussion

Molecular and morphological analyses, as well as geographical distributions, support the description of three new species within the *Phallus indusiatus*-like specimens from Brazil, with partially overlapping distributions. Our results suggest that a great number of species might be hidden within the circum-tropical *P. indusiatus* species concept, since the sequence data obtained from GenBank are clearly polyphyletic with different relationships with other *Phallus* species (Figures 2, 3). In a similar way, several studies have unveiled cryptic fungal diversity hidden within species complexes, especially after the integration of phenotypic, single-DNA and next-generation sequencing (NGS) data (Geml et al. 2006, Jargeat et al. 2010, Kasuya et al. 2012, Kõljalg et al. 2013, Sousa et al. 2017, Accioly et al. 2019). For instance, Geml et al. (2008) revealed that at least eight phylogenetic species are found in the worldwide distribution of *Amanita muscaria* (L.) Lam, with strong intercontinental genetic disjunctions and intracontinental phylogeographic structure. Sousa et al. (2017) revealed four species within the pepper pot *Myriostoma* (Phallomycetidae, Basidiomycota), which has always been considered a monotypic worldwide genus. Long-distance dispersal and cosmopolitanism seems not to be the rule in fungal geographical distribution and, for this reason, there are few species with truly worldwide distributions (Salgado-Salazar et al. 2013). Peay et al. (2016) affirm that climate, environment and dispersal play important roles in shaping fungal communities, where endemism is the most common result in continental and global-scale studies, instead of cosmopolitanism. This becomes clear when analysing the *P. indusiatus* s.l. distribution. As in all gasteroid fungi – basidiomycetes that produce spores inside the fruiting body – this species has a passive mechanism of spore dispersal (statismospory) (Wilson et al. 2011). Phalloid fungi have developed a peculiar spore dispersal mechanism that depends mainly on insects as vectors for dispersal and this factor, together with environmental conditions, should limit *P. indusiatus* s.l. geographical distributions, generating the species mosaic observed here.

Regarding the Brazilian indusiate clade, we suggest that species within this group are, in fact, divergent entities that maintained the general ancestral phenotype (*P. indusiatus* s.l.) throughout their evolutionary history, due to high levels of morphological stasis. This would explain the high frequency of taxonomic uncertainties, which generates a great number of synonyms of *P. indusiatus*. The maintenance of a conserved morphology due to low rates of phenotypic variation has been widely discussed in evolution (Davis et al. 2014). Two main mechanisms have been proposed to explain the small levels of morphological change through time: genetic and developmental constraints may restrict the appearance of phenotypic variation; or there is strong stabilising selection for a phenotype (Lee and Frost 2002, Geml et al. 2008, Davis et al. 2014). In our hypothesis, because the different species in *P. indusiatus* occupy similar niches and, therefore, they are in similar environmental conditions, they are likely to experience similar selective pressures. A similar pattern was found by Mueller et al. (2001) in two disjunct and paraphyletic populations of *Suillus spraguei* (Berk. & M.A. Curtis) Kuntze, that presented no noticeable morphological differences, probably as a result of stabilising selection. For the future, this could be tested for other *Phallus indusiatus*-like species from other continents and alternative methodologies should be applied, such as ancestral state reconstruction.

When studying phalloid species, it is noticeable that macro-characters are more variable than micro-characters. For instance, spores are often cylindrical to bacilloid and smooth throughout the order (except for Gastrosporiaceae), probably as an adaptation for dispersal, since they are dispersed through the gut and do not adhere on the bodies of insects (Tuno 1998, Oliveira and Morato 2000). The presence of rounded crystals in globose cells amongst hyphae of the volva and rhizomorphs was reported for Phallales species (Iofisidou and Agerer 2002), but it is not a commonly used character in species descriptions. Probably these crystals consist of calcium oxalate, as found in other Phallomycetidae species, such as *Gastrosporium simplex* Mattir. and *Geastrum* Pers. (Iofisidou and Agerer 2002, Zamora et al. 2013), but further studies about function and composition in *Phallus* are needed. These crystals are present in most of the species described here, although on different parts: only on the volva of *P. purpurascens*, only on rhizomorphs of *P. squamulosus* and on both volva and rhizomorphs in *P. denigricans*. Further studies are needed in order to evaluate the taxonomic value of the presence of crystals in phalloid fungi. For instance, the presence, shape and the arrangement of oxalate crystals were found to be important characters to delimit species in *Geastrum* (Zamora et al. 2013).

On the other hand, macro-characters, such as the shape, surface and colour of the main structures (receptacle, pseudostipe, indusium, volva and rhizomorphs), are important characters for infrageneric classification (Kreisel 1996). In this study, the phylogenetic clades of *P. indusiatus*-like species were differentiated, based on these features (Table 1), confirming their importance as diagnostic characters. Given that these diagnostic characters are lost once phalloid specimens are dehydrated, it is extremely important that newly described species and new records should be well illustrated with coloured photographs of fresh material. In addition, we believe that molecular data are indispensable for delimiting and describing species in *Phallus*.

**Table 1.** Morphological differences between the new *Phallus* species described here and *Phallus indusiatus.*

	*Phallus denigricans*	*Phallus purpurascens*	*Phallus squamulosus*	*Phallus indusiatus*
**Basidiome development**	Epigeous	Partially epigeous	Epigeous	Initially hypogeous
**Receptacle**	Constricted at the base, pale yellow, prominent apical pore	Conical, thimble-like, flat at the apex, white, strongly reticulated, with an apical pore	Campanulate to thimble-like, with a wide apical pore, strongly reticulated surface	Campanulate, white, reticulated, with an apical pore
**Indusium**	Extending to 2/3 of pseudostipe, poorly developed	Extending to 2/3 of pseudostipe, well developed	Extending to 2/3 of pseudostipe, well developed	Fully developed, extending to the ground
**Volva**	White to blackish, smooth surface or with projections, epigeous	White, becoming purplish, smooth surface, semi-hypogeous	Whitish to pale yellow, squamous surface, epigeous	White, pinkish pigments, hypogeous
**Crystal deposits**	Found on both volva and rhizomorphs of white volva specimens	Found on volva	Found on rhizomorphs	Found on volva
**Basidiospores**	Elongated, 3.6–4.6 × 2.2–2.5 µm	Cylindrical, 4.4–5 × 2.5–3.4 µm	Elongated, 3.5–4.4 × 1.8–2.2 µm	Elongated, 3.6–4.1 × 1.5–2.2 µm

## Supplementary Material

XML Treatment for Phallus denigricans

XML Treatment for Phallus purpurascens

XML Treatment for Phallus squamulosus

XML Treatment for Phallus indusiatus

## Supplementary materials

Supplementary material 1 Table S1 Data type: species data https://binary.pensoft.net/file/344015This dataset is made available under the Open Database License (http://opendatacommons.org/licenses/odbl/1.0/). The Open Database License (ODbL) is a license agreement intended to allow users to freely share, modify, and use this Dataset while maintaining this same freedom for others, provided that the original source and author(s) are credited.Tiara S. Cabral, Bianca D.B. Silva, María P. Martín, Charles R. Clement, Kentaro Hosaka, Iuri G. Baseia

Supplementary material 2 Figure S1 Data type: phylogenetic tree https://binary.pensoft.net/file/344016This dataset is made available under the Open Database License (http://opendatacommons.org/licenses/odbl/1.0/). The Open Database License (ODbL) is a license agreement intended to allow users to freely share, modify, and use this Dataset while maintaining this same freedom for others, provided that the original source and author(s) are credited.Tiara S. Cabral, Bianca D.B. Silva, María P. Martín, Charles R. Clement, Kentaro Hosaka, Iuri G. Baseia

Supplementary material 3 Figure S2 Data type: phylogenetic tree https://binary.pensoft.net/file/344017This dataset is made available under the Open Database License (http://opendatacommons.org/licenses/odbl/1.0/). The Open Database License (ODbL) is a license agreement intended to allow users to freely share, modify, and use this Dataset while maintaining this same freedom for others, provided that the original source and author(s) are credited.Tiara S. Cabral, Bianca D.B. Silva, María P. Martín, Charles R. Clement, Kentaro Hosaka, Iuri G. Baseia
