# Supplementary figures and images for: Behind the veil – exploring the diversity in Phallus indusiatus s.l. (Phallomycetidae, Basidiomycota)
Source: MycoKeys. 2019 Oct 2;58:103–27. doi: 10.3897/mycokeys.58.35324 (PMC6785576; doi:10.3897/mycokeys.58.35324)

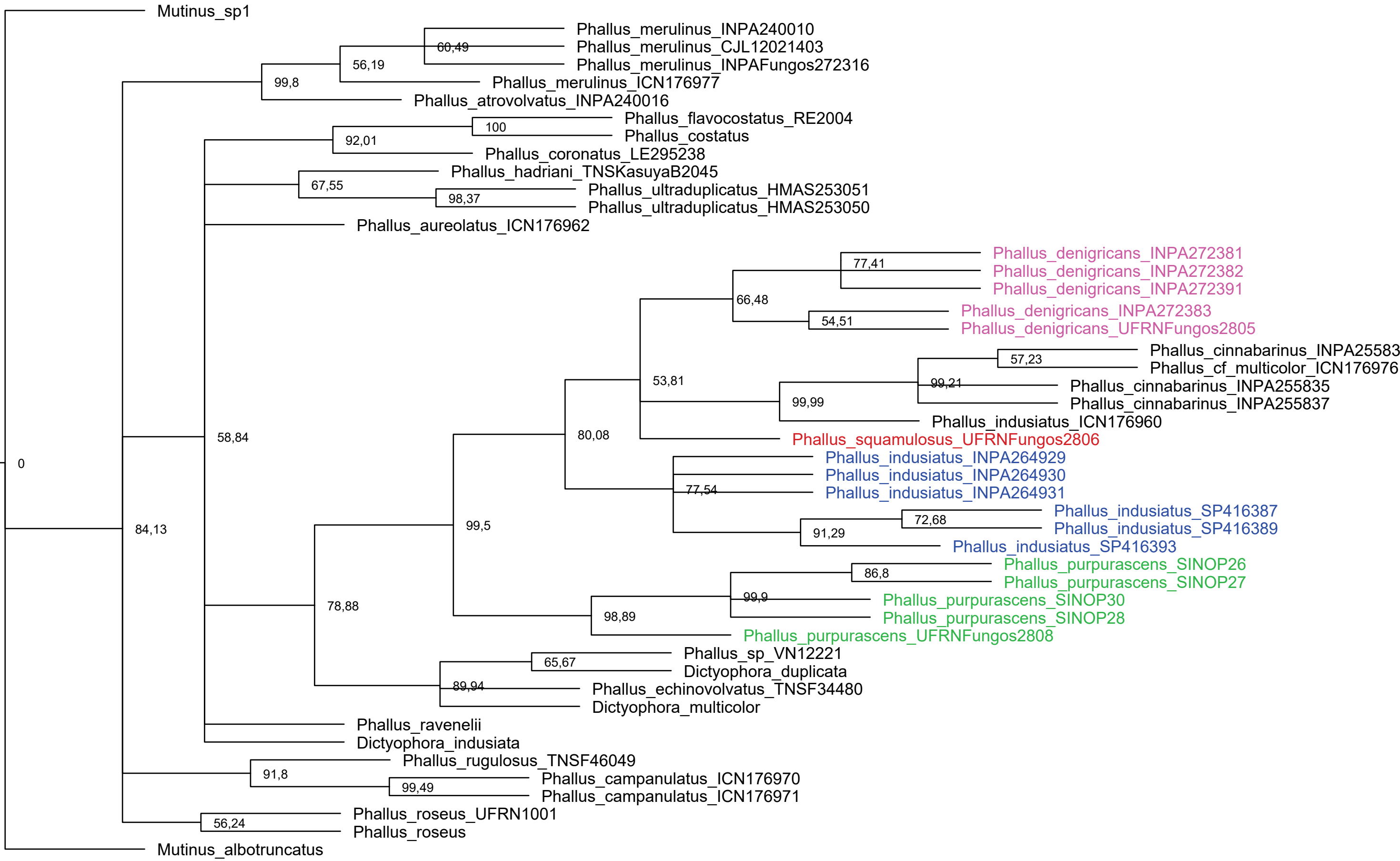

Supplement: Supplementary material 1 [file mycokeys-58-103-s003.pdf]
